# Machine learning and drug discovery for neglected tropical diseases

**DOI:** 10.1186/s12859-022-05076-0

**Published:** 2023-04-24

**Authors:** William Breslin, Doan Pham

**Affiliations:** 1 Department of Mathematics, Computer Science, and Data Science, Pacific University, Forest Grove, OR USA; 2 Dartmouth Institute for Health Policy and Clinical Practice, Geisel School of Medicine, Hanover, NH USA

**Keywords:** Machine learing, Drug discovery, Tropical disease

## Abstract

Neglected tropical diseases affect millions of individuals and cause loss of productivity worldwide. They are common in developing countries without the financial resources for research and drug development. With increased availability of data from high throughput screening, machine learning has been introduced into the drug discovery process. Models can be trained to predict biological activities of compounds before working in the lab. In this study, we use three publicly available, high-throughput screening datasets to train machine learning models to predict biological activities related to inhibition of species that cause leishmaniasis, American trypanosomiasis (Chagas disease), and African trypanosomiasis (sleeping sickness). We compare machine learning models (tree based models, naive Bayes classifiers, and neural networks), featurizing methods (circular fingerprints, MACCS fingerprints, and RDKit descriptors), and techniques to deal with the imbalanced data (oversampling, undersampling, class weight/sample weight).

## Introduction

Leishmaniasis, African trypanosomiasis (sleeping sickness), and American trypanosomiasis (Chagas disease) have been listed in the top list of global burden diseases by the World Health Organization. Leishmaniasis affects 12 million people worldwide, and is considered to be one of the world’s most neglected diseases [1–3]. It is caused by a Trypanosomatid protozoan parasite from the genus *Leishmania*. American trypanosomiasis, also known as Chagas disease, affects more than 7 million people worldwide and is caused by the protozoan parasite Trypanosoma cruzi [2–4]. African Trypanosomiasis, also known as sleeping sickness, affects 300,000 people worldwide and is caused by the parasite *Trypanosoma brucei*.

The only approved therapeutics for the treatment of these diseases are expensive, not very effective and have adverse side effects [5–7]. Thus it is important to keep searching for new treatments. Target-based drug discovery involves “screening a library of compounds against a protein and then optimizing the compounds for potency against the enzyme, selectivity, cellular activity, and pharmacokinetic properties” [8]. As the screening process can involve tens of thousands of compounds, it is often costly and time-consuming. We hope to save time and cut costs by using machine learning in the screening process and only testing compounds in the lab which are predicted to be useful by the models. In this study, we train machine learning models (using data from high-throughout screenings) to predict whether a compound will inhibit a disease-causing parasite. Different models, types of features, and techniques to deal with imbalance datasets are compared. For more on using machine learning in drug discovery for neglected tropical diseases, see [5, 6, 9–12].

## Methods

### Bioassays and data sources

The three datasets used were downloaded from PubChem, a database collecting information on small molecules and datasets on high throughput biological assays and maintained by the National Center for Biotechnology Information.

*Leishmaniasis* The assay used to train models for Leishmaniasis has PubChem AID 1721. The target is the enzyme pyruvate kinase from *Leishmania mexicana*. Of the 292,732 compounds tested, only 1088 are active ($\approx 0.37\%$ active).

*African trypanosomiasis* The assay used to train models for African trypanosomiasis has PubChem AID 485367. The target is the enzyme phosphofructokinase from *T. brucei*. Of the 330,677 compounds tested, only 557 are active ($\approx 0.17\%$ active).

*American trypanosomiasis* The assay used to train models for American trypanosomiasis has PubChem AID 1885. Of the 303,218 compounds tested, only 4394 are active ($\approx 1.45\%$ active).

All three assays were luminescence-based assays. Decreased luminescence indicates inhibition of the target and therefore the corresponding compound may kill the parasite.

### Preprocessing and featurizing

The structure of each compound was downloaded from PubChem in the Structural Data Format (SDF). We then used the python packages DeepChem [13] and RDKit [14] to generate descriptors for each compound.

*Circular fingerprints* Circular fingerprints are a type of hashed fingerprint. All substructures up to a given diameter are enumerated and converted to numeric values using a hash function. These values are used to indicate bit positions in the fingerprints. Thus each compound is represented by a vector of 0s and 1s, with the 1s indicating presence of a substructure. Each position in the vector corresponds to more that one possible substructure (leading to possible “bit collisions”), with longer fingerprints having fewer bit collisions [15]. We used fingerprints of sizes 256 and 1024 in this study.

*MACCS fingerprints* The MACCS (Molecular ACCess System) fingerprints are similar to circular fingerprints because they are vectors of 0s and 1s with each 1 indicating presence of a substructure. MACCS fingerprints are generated using a fixed list of 166 substructures and were developed by Molecular Design Limited, Inc. [16].

*RDKit descriptors* The python package RDKit has a module RDKit.Chem.Descriptors that calculates 208 chemical properties of each compound. A list can be found at [17].

*Concatenations of different descriptors* We also trained our models using concatenations of different features. We used the concatenation of MACCS fingerprints with RDKit features, the concatenation of 1024 fingerprints with RDKit features, and the concatenation of MACCS fingerprints, 1024 fingerprints, and RDKit features.

Since the data is so imbalanced, we used a random stratified splitter to split the entire set of compounds into $80\%$ training set and $20\%$ test set. This preserves the ratio of active to inactive. We used three different splits for training and testing and averaged the results.

### Machine learning models

We trained our machine learning models using the scikit-learn (sklearn) library in python [18] and used the default parameters from sklearn. It should be noted that the performance of the models will most likely improve with hyperparameter tuning. The Keras library was used for neural networks. Early stopping with a patience of 2 was used for the neural network models. A 10% validation set was split from the training set for this.

*Naive Bayes classifiers* The Naive Bayes classifier (NB) is based on Bayes’ Theorem. The “naive” assumption we make is that the descriptors are independent.

*Tree based classifiers* Both of the tree-based models we used are examples of “boosting”. In machine learning, boosting is used to convert many weak models into a single strong model. Gradient Boosted Decision Tree (GBDT) models are based on a method introduced by Friedman [19]. The idea is to use gradient descent to adjust the parameters of a tree in order to decrease a differentiable loss function. The default loss function for GBDT in sklearn is Friedman mean squared error. Adaboost can be thought of as a special case of GBDT in which the loss function is the exponential loss function. It was introduced by Freund and Schapire [20].

*Neural networks* Artificial Neural Networks are models that (very roughly) mimic the complex structure and functioning of the brain [21]. A fully connected neural network can be described by the number of neurons in each hidden layer. We used two different networks, one with a single hidden layer of 100 neurons and one with five hidden layers: 100–100–100–100–20.

### Imbalanced data

The three datasets used to train our models are all very imbalanced. The ratio of active to inactive compounds is very low (ranging from 0.17 to 1.45%), This presents difficulties in both training and evaluation of the models.

#### Measuring success of a model

A common metric for measuring success of a classifier is accuracy score:

$$\begin{aligned} \text{Accuracy} = \frac{TP+TN}{TP+TN+FP+FN}. \end{aligned}$$

If a model were to predict all 330,677 compounds in PubChem AID 485367 are inactive, then the accuracy of that model would be $\approx 0.9983$. However, we would miss all 557 active compounds and we are interested in identifying active compounds. A metric better suited to imbalanced data sets is the balanced accuracy score (BA). It is the average of the true positive rate *TPR* (also known as sensitivity) and true negative rate *TNR* (also known as specificity):

$$\begin{aligned} {\text{BA}} = \frac{TPR+TNR}{2}, \end{aligned}$$

where $TPR=\frac{TP}{P}$ and $TNR=\frac{TN}{N}$. The balanced accuracy score of a model predicting all compounds in PubChem AID 485367 to be inactive would only be 0.5. We use the balanced accuracy as our primary measure of success of our models, but also report accuracy, true positive rate and false positive rate ($FPR = \frac{FP}{N}$).

#### Training models with imbalanced data

We used three techniques to adjust our models to perform better with imbalanced data: oversampling, undersampling, and sample weights.

When we train models using oversampling, active compounds in the training dataset are randomly duplicated until the dataset has the same number of actives and inactives. When using undersampling, a subset with the same size as the set of active compounds is randomly selected from the set of inactive compounds in order to create a balanced training set.

If the sample weights of active compounds are higher than that of inactive compounds, then a misclassified active compound results in a greater contribution to the loss function than a misclassified inactive compound. This also causes the model to predict more active compounds. In our study, all inactive compounds had weight 1. We started with (# of compounds)/(# of active compounds) and experimented to find a range of weights for the active compounds to test.

It should be noted that models trained using oversampling, undersampling, or class weights (with actives weighted more than inactives) also tend to predict more false positives. We stopped increasing the class weights of the actives when the false positive rate exceeded 20%.

## Results and discussion

For each of the three PubChem datasets, we trained five different types of models: Two neural networks (NN1: one hidden layer with 100 neurons, NN2: five hidden layers 100–100–100–100–20), naive Bayes classifier (NB), adaboost tree model (Adaboost), and gradient boosted decision tree (GBDT). Each of these models was trained using five different types of features: Circular fingerprints of size 256 (256FP), circular fingerprints of size 1024 (1024FP), MACCS fingerprints (MACCS), RDKit descriptors (RDKit), and the concatenations (MACCS/RDKit, 1024FP/RDKit, 1024FP/MACCS/RDKit). For each model-feature combination, we trained a model using no imbalance technique, one using oversampling, one using undersampling, and six using different sample weights. Every combination was trained three times using different train-test splits, giving a total of 2520 trained models.

### Models

**Fig. 1:**
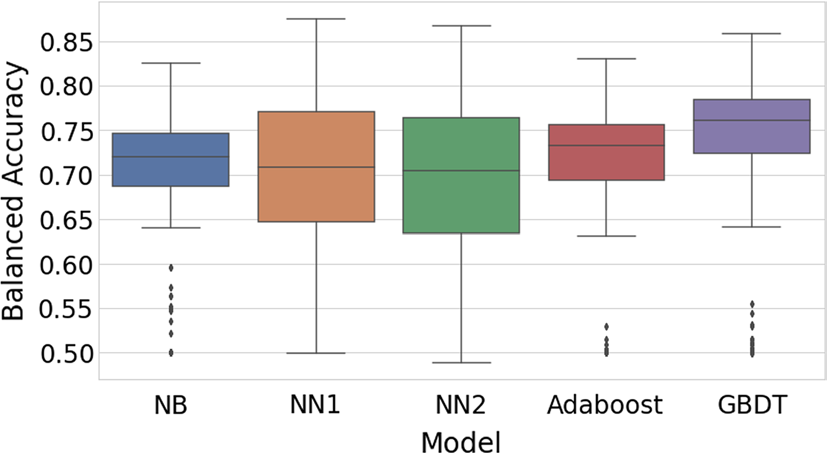
Overall model performance

Figure 1 shows overall performance of the models, with balanced accuracy score shown on the vertical axis. This includes all 2520 models, including training without oversampling, undersampling, or sample weights. Overall, the highest median and mean balanced accuracy score is achieved by GBDT models, with a median balanced accuracy score of 0.762 and mean of 0.738. The neural networks have the highest individual scores but also have higher variance. NN2 has the lowest median balanced accuracy score of 0.705 while NN2 has the second lowest at 0.708.

Tables 1, 2 and 3 show the top model of each type for each of the three assays. The top models for both AID 1721 and AID 1885 are the neural networks. The neural networks had the second and third highest balanced accuracy scores on AID 485367 behind GBDT. The best performance among the three assays is achieved by NN1 on AID 1721 with a balanced accuracy score of 0.875, TPR 0.844, and FPR 0.094.

**Table 1:** Top model performance on AID 1721

Model	BA	TPR	FPR	Accuracy
NB	0.825	0.794	0.143	0.857
GBDT	0.858	0.794	0.077	0.923
Adaboost	0.830	0.835	0.174	0.830
NN1	0.875	0.844	0.094	0.906
NN2	0.868	0.844	0.109	0.891

**Table 2:** Top model performance on AID 1885

Model	BA	TPR	FPR	Accuracy
NB	0.724	0.731	0.281	0.719
GBDT	0.783	0.804	0.238	0.762
Adaboost	0.767	0.811	0.276	0.725
NN1	0.817	0.838	0.203	0.797
NN2	0.813	0.784	0.158	0.841

**Table 3:** Top model performance on AID 485367

Model	BA	TPR	FPR	Accuracy
NB	0.784	0.706	0.139	0.861
GBDT	0.811	0.706	0.084	0.916
Adaboost	0.763	0.706	0.179	0.820
NN1	0.787	0.676	0.102	0.898
NN2	0.810	0.765	0.146	0.854

**Fig. 2:**
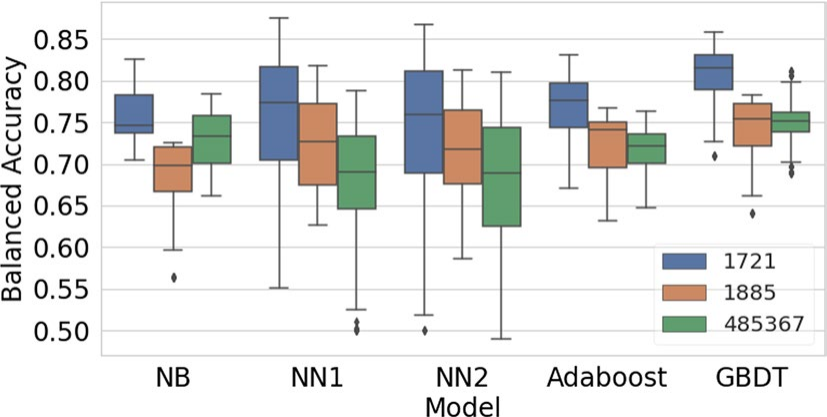
Performance of models on different assays

Figure 2 shows model performance on each of the three bioassays.

### Imbalance techniques

Without oversampling, undersampling, or using sample weights, the performance of all the models is poor. Over 80% of these models have ${\text{TPR}} < 0.1$ and over 44% have ${\text{TPR}} = 0$ (i.e., predicting every compound is inactive). Of the Adaboost models without oversampling, undersampling, or sample weights, over 60% have ${\text{TPR}} = 0$. Only two models trained without oversampling, undersampling, or sample weights have balanced accuracy score above 0.70. Both are Naive Bayes classifiers.

**Fig. 3:**
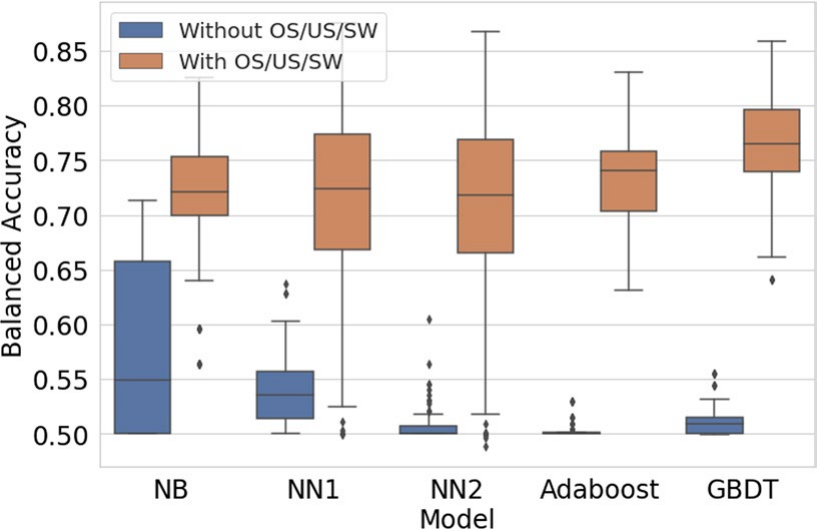
Using imbalance techniques versus not. These plots were created using the entire set of trained models

Figure 3 shows the effect of using imbalance techniques for each model. The performance increases significantly for all models. Table 4 shows mean scores for models trained with oversampling, undersampling, or sample weights versus those trained without oversampling, undersampling, or sample weights.

**Table 4:** Using oversampling (OS), undersampling (US), or sample weights (SW) versus not

Model	Without OS/US/SW			With OS/US/SW		
	BA	TPR	FPR	BA	TPR	FPR
NB	0.581	0.181	0.020	0.724	0.706	0.258
GBDT	0.512	0.023	0.000	0.767	0.716	0.183
Ada	0.503	0.006	0.000	0.735	0.706	0.235
NN1	0.539	0.078	0.000	0.722	0.516	0.071
NN2	0.508	0.017	0.000	0.717	0.523	0.095

For each group, the mean balanced accuracy score (BA), true positive rate (TPR) and false positive rate (FPR) are shown

**Fig. 4:**
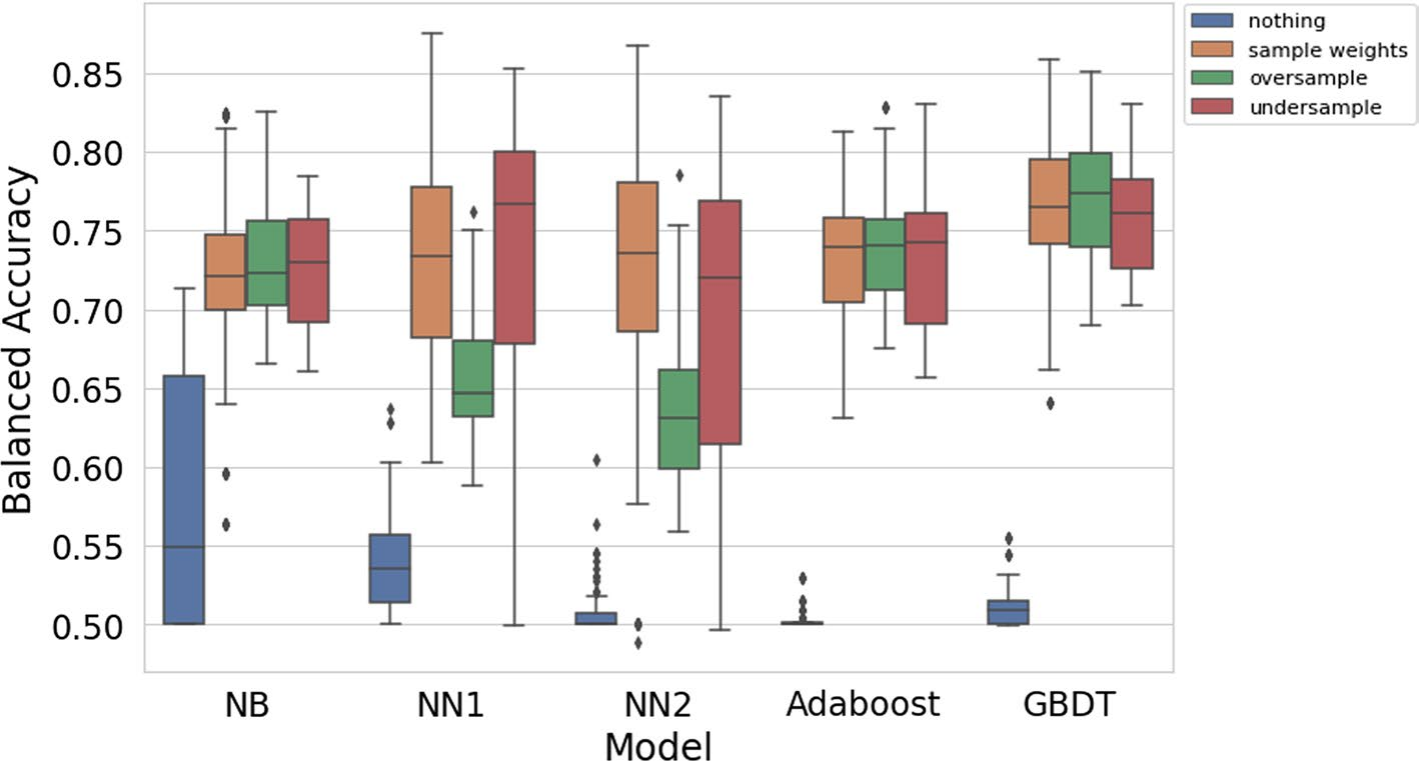
Comparison of imbalance techniques

In Fig. 4, oversampling, undersampling, and class weights are compared for each model type. The boosts in performance of the Naive Bayes models and decision tree classifiers are similar for the three imbalance techniques, while the same is not true for the neural networks. Oversampling does not work as well as using class weights or undersampling for the neural networks.

### Features

**Fig. 5:**
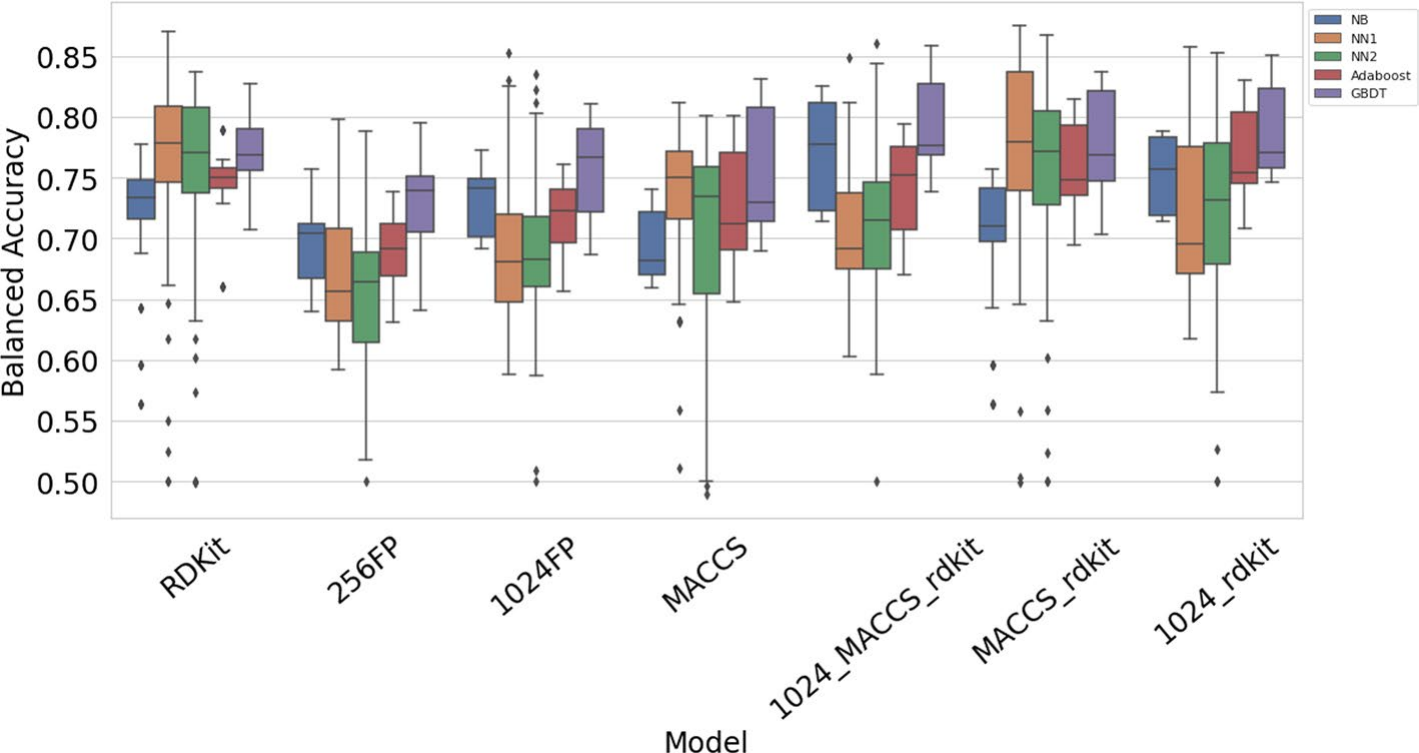
Performance of each feature type when using different models

Figure 5 shows performance of the models, grouped by type of features used. The 1024 circular fingerprints outperform the 256 circular fingerprints, but the results are similar in terms of which models perform better or worse when using circular fingerprints. The 1024FP/RDKit and 1024FP/MACCS/RDKit features have a similar pattern to the circular fingerprints. For these feature types, gradient boosted decision trees are the top performer, followed by naive Bayes classifier and then adaboost. The neural networks were the worst performers when using circular fingerprints, 1024FP/RDKit, or 1024FP/MACCS/RDKit.

However, the neural networks outperformed all other models when using RDKit descriptors, MACCS fingerprints, or the MACCS/RDKit features. It is interesting to note that dropping 1024FP from 1024FP/RDKit or 1024FP/MACCS/RDKit significantly improves performance of the neural networks.

**Fig. 6:**
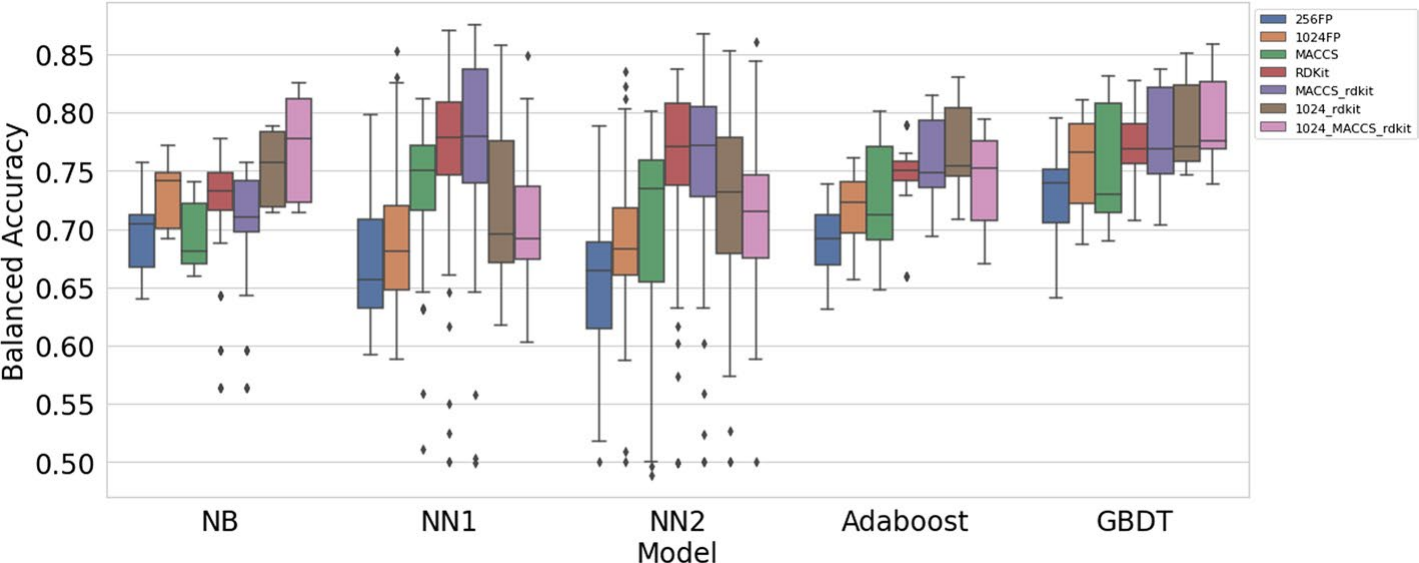
Same scores as shown in Fig. 5, grouped by model

Figure 6 is another look at how the different models perform when using different types of features. The highest median and overall highest balanced accuracy score were obtained when using MACCS/RDKit features with NN1.

Table 5 shows the top balanced accuracy scores by model and feature type.

**Table 5:** Top performing models (highest balanced accuracy score) by model and feature type

Model	Feature type						
	256FP	1024FP	MACCS	RDKit	MACCS/RDKit	1024/RDKit	1024/MACCS/RDKit
NB	0.757	0.772	0.740	0.777	0.757	0.789	0.825
GBDT	0.796	0.811	0.831	0.828	0.837	0.850	0.858
Ada	0.739	0.761	0.801	0.790	0.814	0.830	0.794
NN1	0.798	0.853	0.811	0.870	0.875	0.858	0.849
NN2	0.788	0.835	0.801	0.837	0.868	0.853	0.861

### Training and prediction time

Table 6 shows mean training and prediction times by model. The decision tree models take much longer to train than the neural networks or Naive Bayes classifier. Training the gradient boosted decision trees is very time-intensive, taking 10 times longer than the neural networks and over 4000 times longer than the Naive Bayes classifier. On the other hand, the neural networks take longer during the inference stage.

**Table 6:** Mean training and prediction times by model

Model	Training time (s)	Prediction time (s)
NB	0.31	0.31
GBDT	1172.3	2.23
Ada	212.0	18.7
NN1	72.5	18.0
NN2	72.5	21.3

Table 7 shows mean training and prediction times by feature type. It is not surprising that combined features take the longest for training and predicting, while the times were shortest for MACCS fingerprints (the shortest fingerprints used).

**Table 7:** Mean training and prediction times by feature type

Feature type	Training time (s)	Prediction time (s)
256FP	126.0	9.1
1024FP	440.0	15.2
MACCS	79.6	8.9
RdKit	185.3	8.9
MACCS/RDKit	188.4	8.3
1024/RDKit	469.0	15.6
1025/MACCS/RDKit	704.3	18.7

Using undersampling is fast compared to using class weights or oversampling. The mean training time for a model using undersampling is 3.8 s. The mean training times when using class weights and oversampling are 314.9 s and 551.6 s respectively.

## Conclusion

Neglected tropical diseases affect millions of people worldwide and there is an urgent need to develop new treatments. Identifying compounds that interact with appropriate drug targets is an important part of the drug discovery process. In this project, we trained machine learning models to predict biological activity of drug compounds and compared the results using test data. We used publicly available data to train and test our models. All three datasets are very imbalanced with many more inactive compounds than active. We compared different models, feature types, and ways to deal with the data imbalance.

Without using oversampling, undersampling, or class weights, the models did not perform well, with balanced accuracy scores not much higher than 0.5. After either using class weights or oversampling, many of the models were quite accurate. Oversampling, undersampling, and class weights resulted in a similar boost in performance for the Naive Bayes, Adaboost, and gradient boosted decision tree models, while oversampling did not perform as well as undersampling and class weights for the neural networks. Undersampling has the additional advantage of using less time and memory.

The gradient boosted decision tree models were consistently accurate while using different feature types, but took far longer to train than the other models. The Naive Bayes classifiers have an acceptable performance and are much faster to train. Several of the best single models were neural networks, but the neural networks had a larger range of balanced accuracy scores and more worse performing models.

Overall, training with concatenations of the RDKit features, 1024 fingerprints, and MACCS fingerprints yielded more accurate models than using a single type of feature, but also lead to longer training times. The neural networks performed better when using only MACCS/RDKit features while other models performed better when using all three 1024FP/MACCS/RDKit features. Among the single feature types, the RDKit descriptors produced the best models.

These models can used to prioritize compounds in screening experiments and we hope such techniques lead to a more efficient drug discovery process.

## Data Availability

The unprocessed datasets are available from PubChem (https://pubchem.ncbi.nlm.nih.gov/): AID 1721, AID 1885, AID 485367. The code and processed data will be available for research purposes upon request. Interested readers can contact William Breslin at breslin@pacificu.edu.
